# Proliferation Cycle Transcriptomic Signatures are Strongly associated With Gastric Cancer Patient Survival

**DOI:** 10.3389/fcell.2021.770994

**Published:** 2021-12-01

**Authors:** Jianwen Hu, Yanpeng Yang, Yongchen Ma, Yingze Ning, Guowei Chen, Yucun Liu

**Affiliations:** ^1^ Department of General Surgery, Peking University First Hospital, Beijing, China; ^2^ Department of Endoscopy Center, Peking University First Hospital, Beijing, China

**Keywords:** the cancer genome atlas, gene expression omnibus, proliferation, cell cycle, cluster, gastric cancer

## Abstract

Gastric cancer is one of the most heterogeneous tumors with multi-level molecular disturbances. Sustaining proliferative signaling and evading growth suppressors are two important hallmarks that enable the cancer cells to become tumorigenic and ultimately malignant, which enable tumor growth. Discovering and understanding the difference in tumor proliferation cycle phenotypes can be used to better classify tumors, and provide classification schemes for disease diagnosis and treatment options, which are more in line with the requirements of today’s precision medicine. We collected 691 eligible samples from The Cancer Genome Atlas (TCGA) and Gene Expression Omnibus (GEO) database, combined with transcriptome data, to explore different heterogeneous proliferation cycle phenotypes, and further study the potential genomic changes that may lead to these different phenotypes in this study. Interestingly, two subtypes with different clinical and biological characteristics were identified through cluster analysis of gastric cancer transcriptome data. The repeatability of the classification was confirmed in an independent Gene Expression Omnibus validation cohort, and consistent phenotypes were observed. These two phenotypes showed different clinical outcomes, and tumor mutation burden. This classification helped us to better classify gastric cancer patients and provide targeted treatment based on specific transcriptome data.

## Introduction

From a global perspective, gastric cancer incidence is relatively high, ranking fourth (Sung et al., 2021). The incidence and mortality of gastric cancer ranks second among malignant tumors in China (Zheng et al., 2019). Surgery combined with radiotherapy and chemotherapy has contributed to the longer survival of gastric cancer, but at present it is only beneficial to some patients (Wöhrer et al., 2004; Liu et al., 2019). The long-term prognosis is an urgent clinical problem to be solved.

Maintaining proliferation signals and avoiding growth inhibitory factors are two important signs that make cancer cells tumorigenic and eventually malignant, thereby enabling tumors growth (Hanahan and Weinberg, 2011; Ateshian et al., 2012). Normal tissues gingerly regulate the generation and release of pro-growth signals, which indicates cells entry and progression through the cell growth and division cycle, thus making sure that the steady state of cell numbers and maintaining normal tissue structure (Sulić et al., 2005; Lemmon and Schlessinger, 2010; Ateshian et al., 2012). Cancer cells can acquire the ability to maintain proliferation signals through a variety of ways: they can produce growth factor ligands and respond through homologous receptors expression, leading to autocrine proliferation stimulation (Perona, 2006; Hynes and MacDonald, 2009; Lemmon and Schlessinger, 2010; Witsch et al., 2010). In addition, cancer cells can send signals to stimulate normal cells in the tumor-associated stroma, which can offer various growth factors to the cancer cells (Bhowmick et al., 2004; Cheng et al., 2008). The destruction of the negative feedback mechanism that inhibits proliferation signals is another mechanism for cancer development. The deficiencies of these feedback mechanisms can enhance proliferation signals (Cabrita and Christofori, 2008; Wertz and Dixit, 2010). In addition to inducing and maintaining positive growth stimulating signals, cancer cells must also bypass powerful procedures that negatively affect cell proliferation (Amit et al., 2007; Cabrita and Christofori, 2008; Hanahan and Weinberg, 2011). Insufficient understanding of the microenvironment of gastric cancer proliferation cycle may be the main reason for the disappointing results. The rapid development of transcriptomics has made it possible to systematically explore the temporal heterogeneity of genomics in gastric cancer.

Discovering and understanding the differences in tumor proliferation cycle phenotypes can better classify tumors, provide classification schemes for disease diagnosis and treatment, and better meet the requirements of precision medicine today. At the same time, it is conducive to find more sensitive and specific biomarkers, help predict the prognosis of tumors, and develop more effective anti-tumor drugs. The purpose of this study is to use open cohorts to identify the molecular subtypes of gastric cancer, identify the relationship between each cluster and clinical data, determine the unique molecular characteristics of each cluster, and establish a corresponding classifier. In the same time, class gene labels and classifiers can be obtained in this way to predict the classification of new samples, achieve the purpose of identifying cancer subtypes in new samples, establish targeted treatment plans for individuals, reduce the mortality of cancer patients, and improve the mortality of patient rate and improve living standards.

In this study, we collected qualified samples from TCGA and GEO cohorts, and combined with transcriptome data to explore different heterogenous proliferation cycle phenotypes, and further investigated the potential mechanism of each proliferation phenotype.

### Patient Datasets

In this study 691 gastric cancer patients (334 from TCGA-STAD sequencing data and 357 from GEO chip data) were included. Transcriptome sequencing data and clinical information were downloaded from the TCGA database (https://portal.gdc.cancer.gov/) as a training cohort. The chip data and the corresponding clinical information were downloaded from the GEO database (https://www.ncbi.nlm.nih.gov/geo/) as the validation cohort. These samples had complete transcriptome data, clinical data and follow-up data and non-zero survival time at the same time in the TCGA-STAD cohort. Correspondingly, we also selected GEO Chip data (GSE84433) with a large enough data volume and relatively complete clinical data and follow-up data as the validation cohort.

### Transcriptome-Based Subtypes Identification

The R “ConsensusClusterPlus” package was applied to cluster gene transcriptome data. Two main subtypes were identified by hierarchical clustering of 5,000 genes with highly variable expression [top 5,000 of Median Absolute Deviation (MAD) of gene value]. 80% of the items to resample, 50 resamplings and the maximum evaluation K of 10 were used for gene expression clustering. The cumulative distribution function (CDF) and consensus heat map were applied to evaluate the best K.

### Selection of Gene Signatures

The differentially expressed genes were identified with the statistically difference of |log_2_FC| > 0.58496 and false discovery rate [FDR] <0.05 between subtype A (Sub A) and subtype B (Sub B) firstly. A univariate cox regression analysis was performed to determine the genes with prognostic significance. Subsequently, the method of lasso regression was used to determine the target genes that have the greatest weight on the prognosis by the R package “glmnet” (Friedman et al., 2010) (https://www.jstatsoft.org/v39/i05/). The influence of gene expression on prognosis was weighed by the enter method-based multivariate cox analysis. The prognostic risk signature was built by combining the values of identified gene expression and their corresponding regression coefficients (β value). The median of risk score was set as a cutoff value and divided gastric cancer into high and low risk groups. The differences of overall survival between high and low risk groups were assessed by the R “survival” package (version 3.2–7, https://CRAN.R-project.org/package=survival).

### Bioinformatics Analysis

Principal component analysis (PCA) was used to detect expression differences between groups with the R package “princomp” (Zhou et al., 2018). Function annotation of difference genes between groups was analyzed by Gene Ontology (GO) analysis (Huang et al., 2009). Gene set enrichment analysis (GSEA) was performed to determine statistically different gene sets (Mootha et al., 2003; Subramanian, 2005). GO analysis and KEGG analysis conducted by R clusterProfiler R package (3.14.3 version) (Carlson, 2015). The R package “pROC” was used to analyze the receiver operating characteristic (ROC) curve to predict the overall survival (OS). The catalogs of genes related to each stage of the cell cycle were obtained from GO (http://geneontology.org/), KEGG (https://www.genome.jp/kegg/) database and references at the same time. The level of infiltration of different immune cells in the TCGA-STAD and GEO data was quantified by the “CIBERSORT” R package (Newman et al., 2015) with LM22 features and 1,000 permutations. ESTIMATE algorithms assessed the immune and matrix content (immune and stromal scores) of each TCGA-STAD and GEO sample (Yoshihara et al., 2013). The results were displayed in the form of heatmaps and histograms.

### Statistical Analysis

SPSS 24.0 (IBM, Chicago, Illinois, United States) and R software (version 3.6.3; http://www.r-project.org/) were used to conduct all statistical analysis. All visualizations were implemented by R software. Kaplan-Meier analysis was used to evaluate survival differences between the groups with log-rank test. Student’s *t*-test was used to calculate differences in molecular expression, stromal score, immune score and ESTIMATE score between the two subtypes. *p* < 0.05 was considered as statistically significant.

## Results

### Gastric Cancer Differentiates Into Two Molecular Subgroups

**FIGURE 1:**
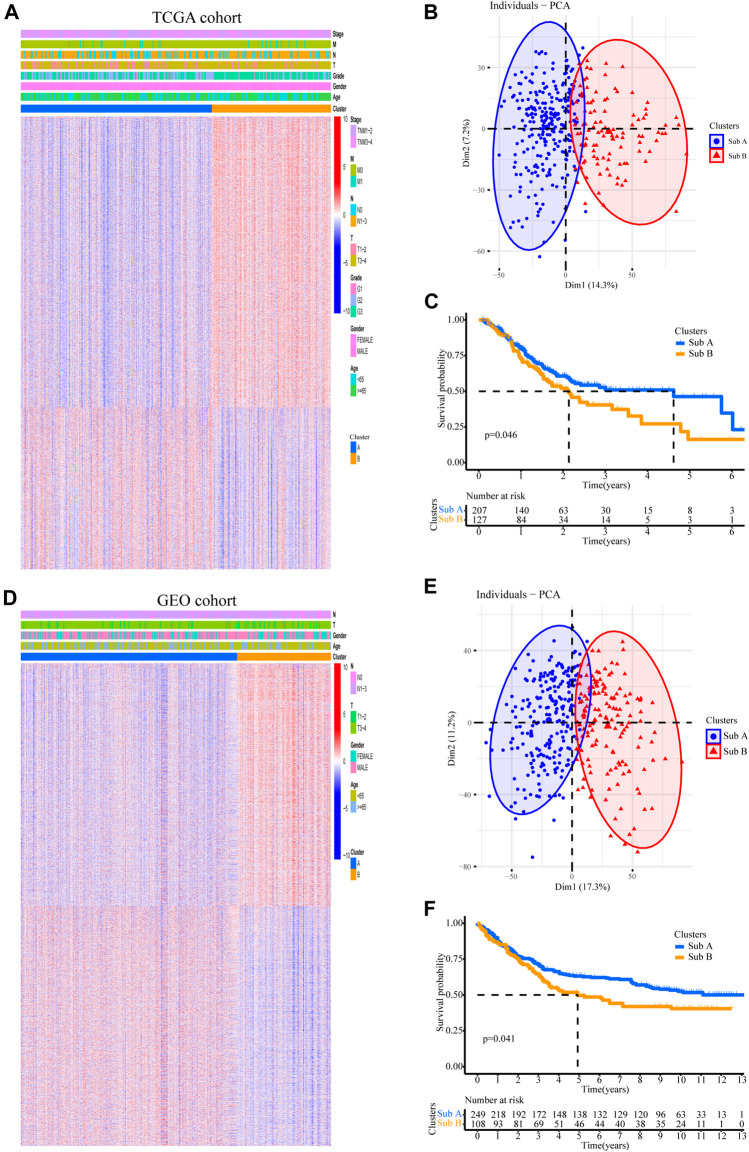
Two subtypes with different prognosis in gastric cancer were identified by unsupervised cluster analysis. **(A)** Two subtypes defined by 5,000 genes with highly variable expression are plotted as a heatmap. Genes with MAD value in the top 5,000. **(B)** Principal component analysis (PCA) revealed differences between the two subtypes in TCGA cohort. **(C)** Kaplan-Meier survival plot for two subtypes in TCGA cohort. **(D)** The gene sequence of the TCGA training set was applied to the GEO validation cohort. **(E)** PCA analysis revealed differences between the two subtypes in GEO validation cohort. **(F)** Kaplan-Meier survival plot for two subtypes in the GEO validation cohort.

In order to stratify patients with gastric cancer, we obtained 334 samples of sequencing data from the TCGA-STAD database, using unbiased methods and consistent clustering of gene expression profiles. Through cumulative distribution function (CDF) curve and consensus matrix evaluation (Supplementary Figure S1), we hierarchically clustered 5,000 highly variable expression genes (top 5000 MAD of gene value) and identified two main subtypes (Figure 1A). Principal component analysis (PCA) plot found significant differences in the expression profiles of the two subtypes (Figure 1B). Group members of these two subtypes were associated with different molecular and survival characteristics. Compared with Sub A, the clinical outcome of Sub B patients was significantly worse. In contrast, Sub A had a longer overall survival than Sub B group (Figure 1C).

357 independent gastric cancer expression profiles were obtained from the GEO (GSE84433) cohort to evaluate the reproducibility of subtypes. Applying the similar genes rankings from the training set (5,000 available genes) to the validation set can clearly replicate the identified subgroups in the TCGA cohort (Figure 1D). PCA also confirmed the difference in gene expression profiles between these two subtypes (Figure 1E). Similarly, survival analysis showed that Sub B had a shorter overall survival time than Sub A (Figure 1F).

### Differences in Biological Functions of the Two Subtypes

**FIGURE 2:**
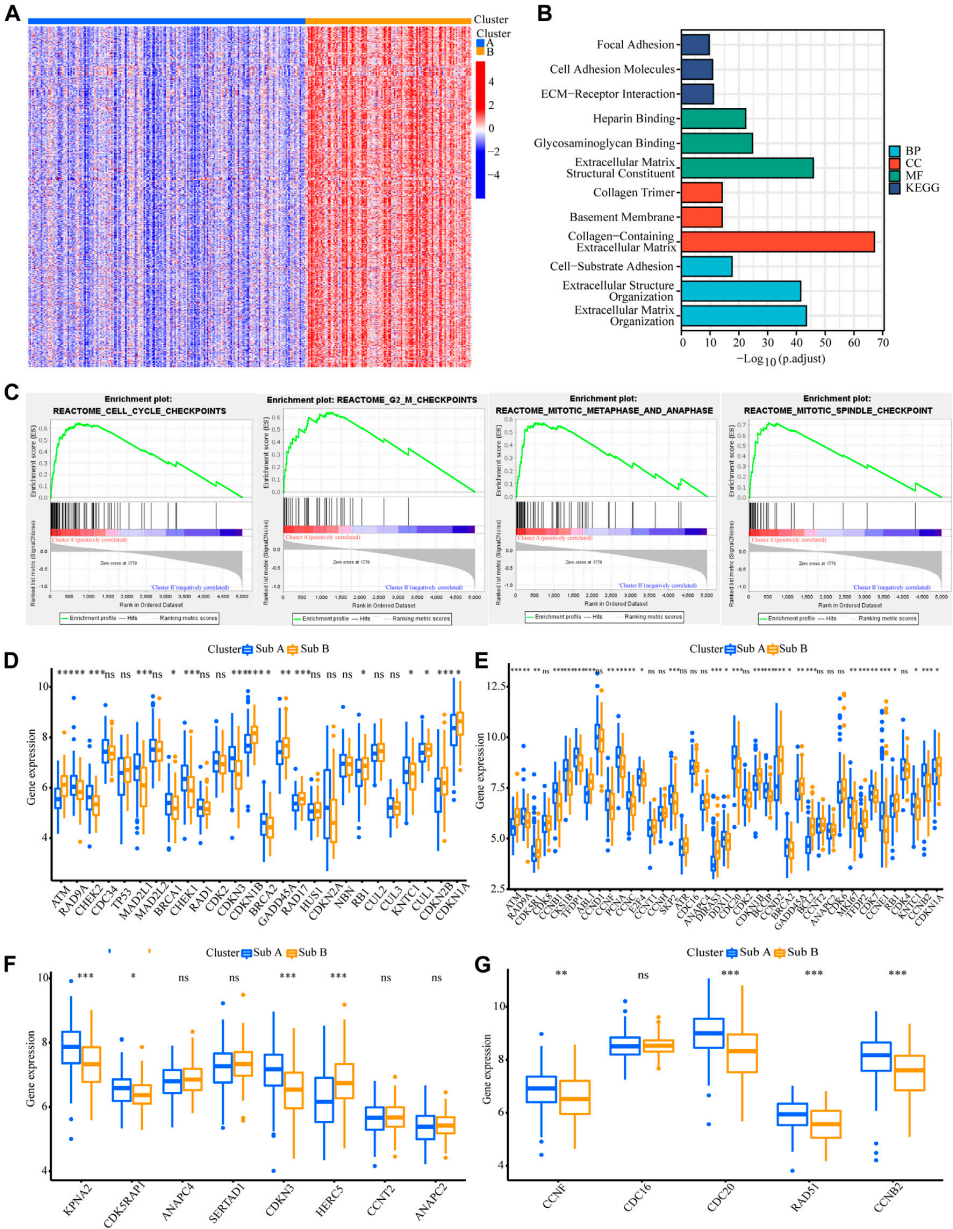
Analyses of the biological functions of the two subtypes in the TCGA cohort. **(A)** The heatmap showed DEG between Sub A and Sub B. **(B)** GO and KEGG analyses on upregulated genes in Sub A vs Sub B in TCGA cohort. **(C)** GSEA analysis of the two subtypes in the TCGA cohort. **(D–G)** A box plot showed the difference in the expression of cell cycle checkpoint **(D)**, cell cycle G2/M phase transition **(E)**, negative regulation of nuclear division **(F)** and regulation of spindle checkpoint **(G)** between the two subtypes. *p < 0.05; **p < 0.01; ***p < 0.001.

**FIGURE 3:**
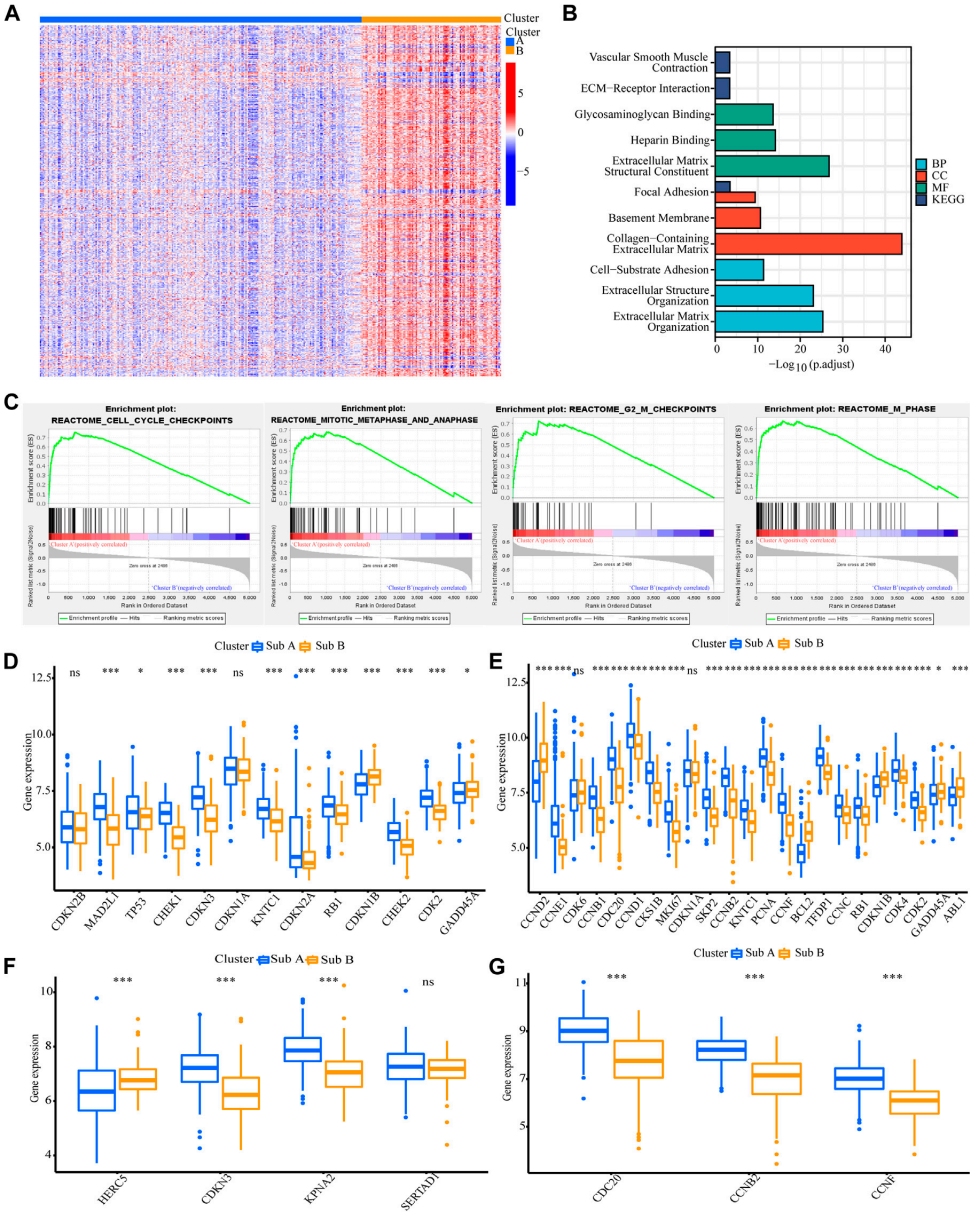
Analyses of the biological functions of the two subtypes in the GEO cohort. **(A)** The heatmap showed the DEG between Sub A and Sub B. **(B)** GO and KEGG analyses on upregulated genes in Sub A vs Sub B in GEO cohort. **(C)** GSEA analysis of the two subtypes in the GEO cohort. **(D–G)** A box plot showed the difference in the expression of cell cycle checkpoint **(D)**, cell cycle G2/M phase transition **(E)**, negative regulation of nuclear division **(F)** and regulation of spindle checkpoint **(G)** between the two subtypes. *p < 0.05; **p < 0.01; ***p < 0.001.

We also analyzed the functional background of the two gastric cancer subtypes. Figure 2A showed the results of cluster analysis by differential gene expression between the two groups. GO and KEGG analyses with TCGA cohort showed that the up-regulated genes in Sub A vs Sub B were enriched in cell cycle, cell cycle G2/M phase transition, cell cycle checkpoint and negative regulation of chromosome separation (Figure 2B). GSEA further showed that the up-regulated genes in Sub A vs Sub B were mainly enriched in cell cycle checkpoints, G2/M checkpoints, mitotic metaphase and anaphase, and mitotic spindle checkpoint (Figure 2C). Differential gene expression analysis showed cell cycle checkpoint (Figure 2D), cell cycle G2/M phase transition (Figure 2E), negative regulation of nuclear division (Figure 2F) and regulation of spindle checkpoint (Figure 2G) related genes were highly expressed in Sub A samples. The same enrichment trend also appeared in GEO cohort (Figures 3A–G).

GO and KEGG analyses on genes that were upregulated in Sub B vs Sub A in TCGA cohort showed that extracellular matrix structural constituent, collagen-containing extracellular matrix and extracellular matrix organization were enriched (Supplementary Figure S2A). The same enrichment trend also appeared in GEO cohort (Supplementary Figure S2B). Since the differential genes of Sub B vs Sub A were mostly clustered in extracellular matrix related pathways, we analyzed the infiltration of stromal cells in Sub A and Sub B. We implemented the CIBERSORT and ESTIMATE algorithms to quantify the activity or enrichment level of immune cells in gastric cancer tissues. The results showed that the stromal cell infiltration and score, the degree and score of immune cell infiltration, and the overall extracellular matrix infiltration score of Sub B subtypes were higher than Sub A in TCGA and GEO cohorts (Supplementary Figure S3 and S4).

In order to understand the significance of Sub A and Sub B classification, we put our findings in the context of well-recognized molecular subtypes of gastric cancer (EBV, MSI, GS, and CIN) (Sohn et al., 2017) and the results were shown in Supplementary Figure S4. We made the survival curves of Sub A and Sub B among the four recognized classification subtypes, and they were not statistically significant. However, Sub A tended to have a longer survival than Sub B in the MSI and CIN subtypes (Supplementary Figure S5C,D). EBV subtype had better prognosis and overall survival (Sohn et al., 2017) and there were more EBV subtypes in the Sub A sample (Supplementary Figure S5E). At the same time,Kaplan-Meier survival analysis was performed on Sub A and Sub B in different TCGA and GEO clinical characteristics (Supplementary Figure S6, S7). Sub A samples had a better survival trend than Sub B samples. A quantitative analysis of different characteristics enriched in Sub A and Sub B in TCGA and GEO cohorts was also conducted. The results showed that Sub B samples had a higher proportion of well-recognized clinical indicators with poor relative prognosis (Supplementary Figure S8). These illustrated the feasibility of the classification method to a certain extent.

### Analysis of Differences in Cycle Control Molecules

Since the up-regulated molecular functions in Sub A vs Sub B were enriched in cell cycle-related pathways, the difference genes in cyclin expression between the two subtypes were analyzed. Cell cycle checkpoint, cell cycle G2/M phase transition, negative regulation of nuclear division and regulation of spindle checkpoint related gene expression were analyzed between the two subtypes. Sub A had a higher expression of negative cell cycle control proteins than Sub B in TCGA cohort (Figures 2D–G). Sub A highly expressed CHEK2, MAD2L1 and SFN genes, which were two typical cell cycle checkpoint genes, and their high expression can reduce cell proliferation (Stolz et al., 2011; Lewinska et al., 2017; Marima et al., 2021) (Figure 2D). Sub A highly expressed CDKN3 and CHEK1 which were cell cycle suppressor genes (Lee et al., 2000; Liu et al., 2000) belonging to the G2 and G2/M phase-related proteins (Figure 2F). Sub A samples expressed BUB1and PLK1 higher than sub B. BUB1and PLK1 are typical genes that negative regulate nuclear division (Fukagawa, 2008; de Cárcer et al., 2018). The same performance was also verified in GEO data (Figures 3D–G).

### The Correlation Between Tumor Burden Mutation and the Two Subgroups

**FIGURE 4:**
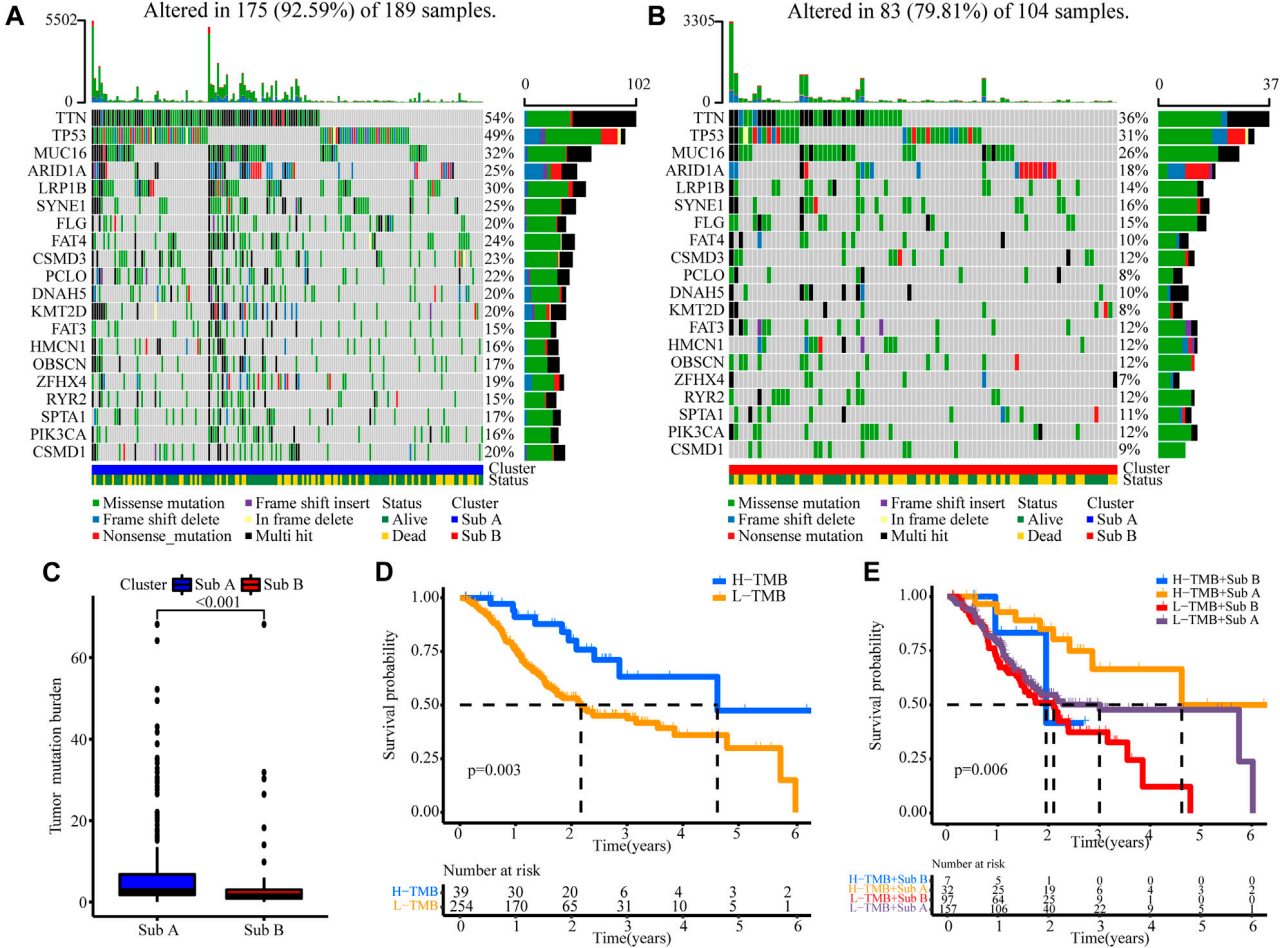
The correlation between the TMB and clusters in TCGA cohort. **(A)** TMB analysis in the Sub A groups. **(B)** TMB analysis in the Sub B groups. **(C)** Difference analyses of TMB between Sub A and Sub B groups. **(D)** Kaplan-Meier curves for low and high TMB groups of the TCGA cohort. **(E)** Kaplan-Meier curves for patients in the TCGA cohorts stratified by both TMB and clusters.

Next, we analyzed tumor burden mutation (TMB) from the TCGA cohort to explore the differences in genomic changes between the two subtypes. Figures 4A,B showed the TMB situation of Sub A and Sub B. Sub A displayed more deletion regions, such as TTN, TP53, MUC16, ARID1A, LRP1B, SYNE1 and FAT4 (Figures 4A,B). As shown in Figure 4C, the TMB of the Sub A group was significantly higher than that of Sub B group (*p* < 0.001). Kaplan-Meier survival analysis showed that patients with high tumor burden mutation (H-TMB) had better overall survival than the low tumor burden mutation (L-TMB, *p* = 0.003, Figure 4D). Considering the prognostic value of TMB and clusters, we next evaluated the synergy effect of these indicators in the prognostic stratification of TCGA-STAD. Stratified survival analysis revealed that the TMB status did not affect cluster-based predictions. Sub A and Sub B showed significant survival differences in both high and low TMB subgroups (*p* = 0.006; Figure 4E). Overall, these results indicated that the stratification may be an underlying predictor that is independent of TMB.

### Identification of Prognostic Characteristics Related to Gastric Cancer Classification and Construction of Clinical Prognostic Risk Model

#### Screening of Differential Molecules With Survival Prognostic Value

**FIGURE 5:**
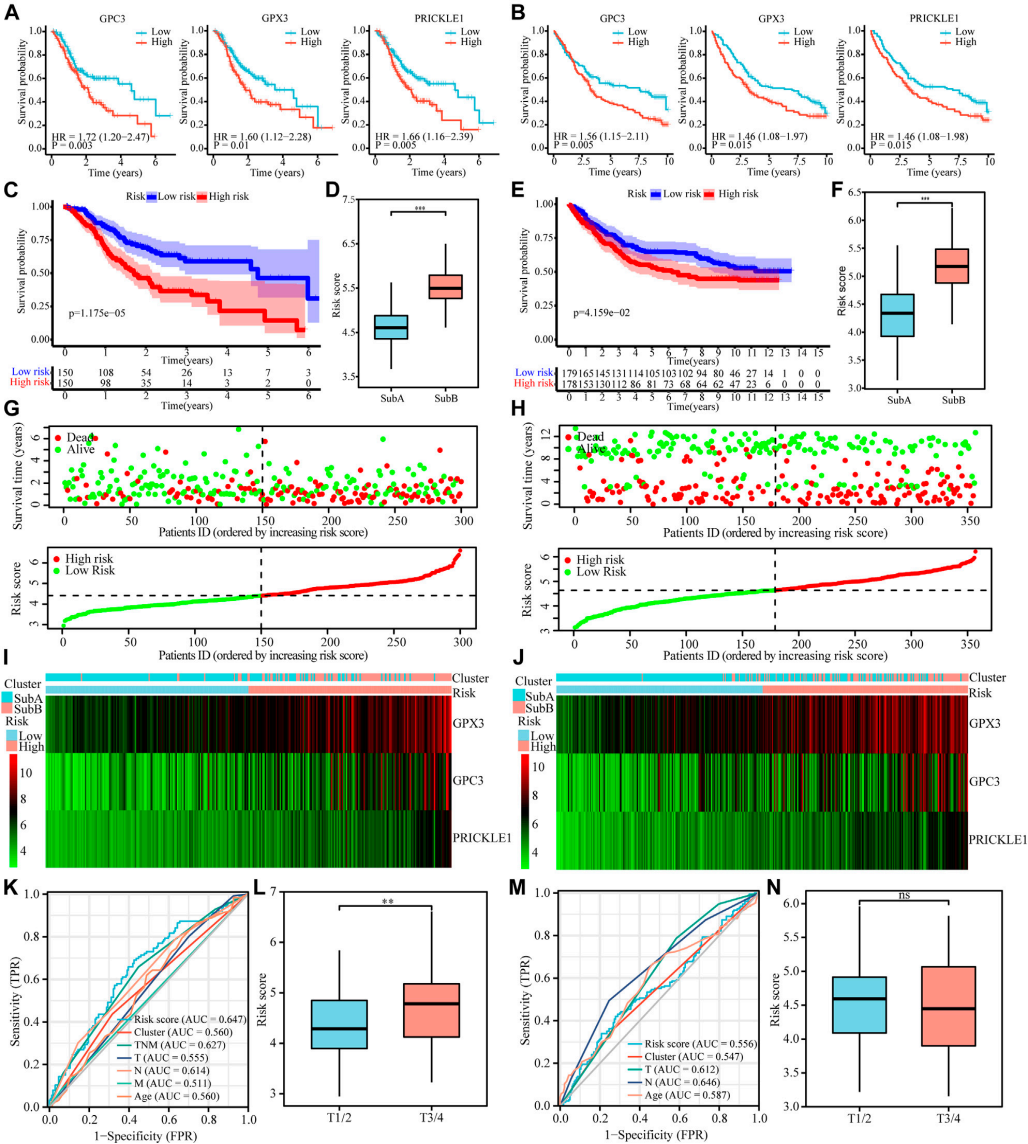
Cox proportional hazards model was used to identify the prognostic signature. (A and B) Kaplan-Meier curve plot for key prognostic genes of TCGA **(A)** and GEO **(B)** cohorts. **(C and E)** Kaplan-Meier curve plot for key prognostic genes-based risk model of TCGA **(C)** and GEO **(E)** cohorts. **(D and F)** Risk score of the two subtypes in TCGA **(D)** and GEO **(F)** cohorts. **(G and H)** Distribution and current status of gastric cancer risk scores in TCGA and GEO cohorts, which indicated that high-risk score was related to poor prognosis. **(I and G)** Heatmaps showed that the degree of key prognostic genes expression in different risk score and the two subtypes in TCGA **(I)** and GEO **(G)** cohorts. **(K and M)** ROC of risk score prediction model on TCGA **(K)** and GEO **(M)** cohorts. **(L and N)** Survival curves of gastric cancer patients with combinations of risk score and T stage in the TCGA **(L)** and GEO **(N)** cohorts.

**FIGURE 6:**
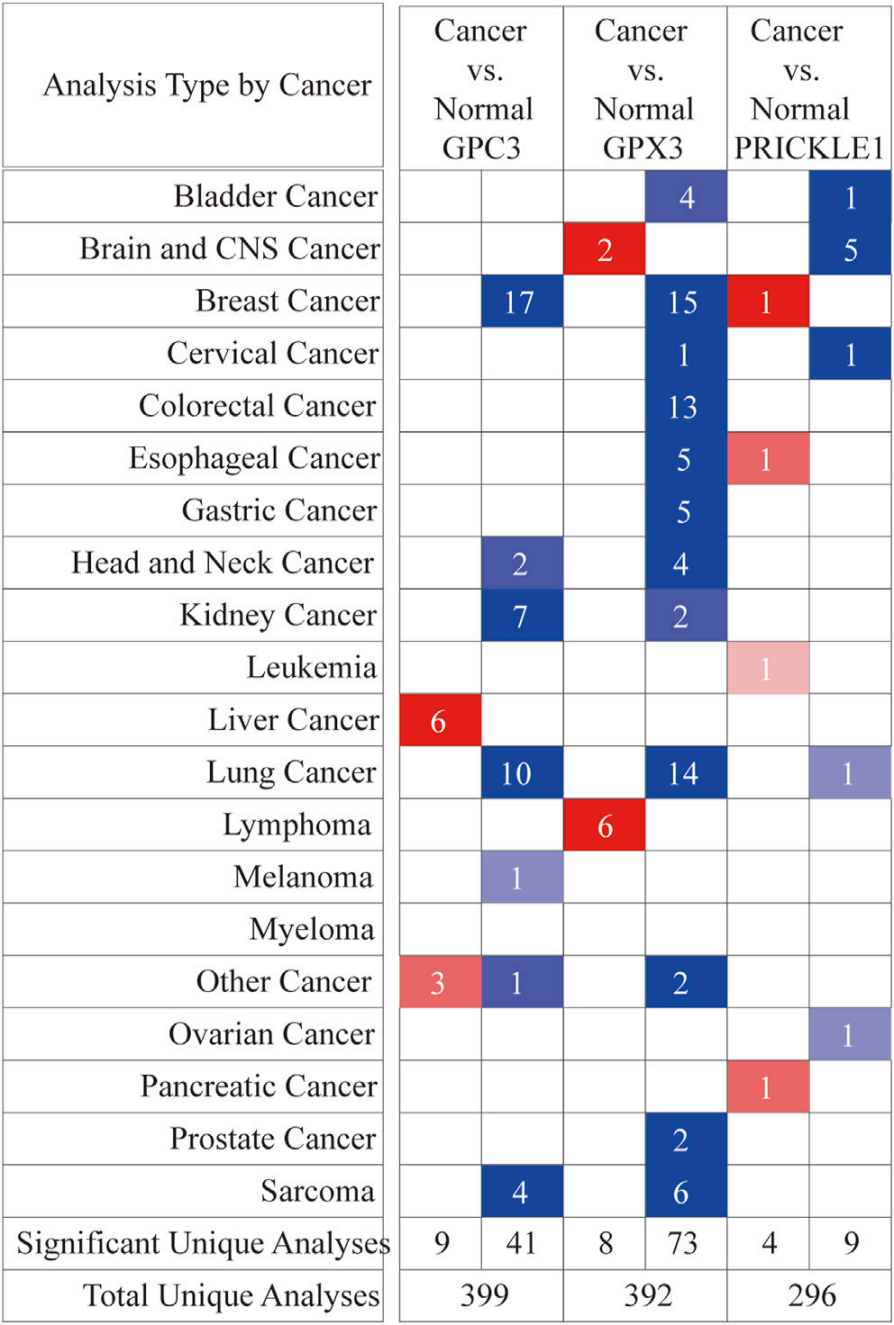
Differential expression of three signature genes in cancer and adjacent tissue in 20 tumors at the ONCOMINE database. Student’s t test was used to compare the differences in mRNA expression. Red represented overexpression and blue represented low expression. The darker the color, the more obvious the difference in gene expression.

We used the lasso regression method to screen 236 differential genes of the Sub A and Sub B with prognostic significance. The corresponding clinical information was shown in Table 1. The remaining three molecules in the final model that had a greater impact on the prognosis were GPC3, GPX3 and PRICKLE1. Survival analysis showed that high expression of these three molecules showed worse prognosis in TCGA and GEO cohorts (Figures 5A,B) which indicated that the three selected molecules may have the potential to predict prognosis. Therefore, we combined clinical indicators and the expression of the three molecules, and applied cox multivariate regression analysis to determine the weight of the three molecule’s influence on the prognosis, thereby constructing a prognostic risk prediction model. We combined the expression value of the identified genes and the weighting of the regression coefficient (β value) to construct the prognostic risk characteristics respectively. Risk score = GPC3*0.142 + GPX3 *0.262 + PRICKLE1*0.366. The median of risk score was set as a cutoff value and divided gastric cancer patients into high and low risk groups. Kaplan-Meier survival analysis illustrated that high-risk group had worse prognosis for overall survival (*p* < 0.05, Figures 6C,E) in TCGA and GEO cohorts. Enter method-based cox multivariate regression was performed to evaluate the prognostic influence of the risk signature combined with other clinic pathological features. The results showed that the risk score was one of the independent risk factors for overall survival among gastric cancer patients (*p* < 0.05, Table 2).

**TABLE 1:** Clinical characteristics of patients with gastric cancer in TCGA and GEO database.

	TCGA cohort	GEO cohort
Characteristics	No. of patients (%)	No. of patients (%)
**Age**		
<65	129 (43.0)	229 (64.2)
≧65	171 (57.0)	128 (35.8)
**Gender**		
Female	110 (36.6)	115 (32.2)
Male	190 (63.4)	242 (67.8)
**Grade**		
G1&G2	111 (37.0)	
G3	189 (63.0)	
**T**		
T1&2	77 (25.6)	46 (12.8)
T3&4	223 (74.3)	311 (87.2)
**N**		
N0	89 (29.6)	71 (19.8)
N1&2&3	211 (70.3)	286 (80.1)
**M**		
M0	280 (93.3)	
M1	20 (6.4)	
**TNM**		
1&2	139 (46.3)	
3&4	161 (53.6)	

**TABLE 2:** Cox regression analysis of risk score in TCGA and GEO cohorts.

TCGA cohort					GEO cohort			
Univariate cox			Multivariate cox		Univariate cox		Multivariate cox	
Characteristics	HR (95%CI)	*p* value	HR (95%CI)	*p* value	HR (95%CI)	*p* value	HR (95%CI)	*p* value
**Age**	1.019 (1.002,1.037)	0.031	1.034 (1.015,1.054)	0.000	1.019 (1.005,1.033)	0.009	1.026 (1.011,1.040)	0.000
**Gender**								
Male vs Female	1.439 (0.980,2.113)	0.063	1.597 (1.083,2.356)	0.018	1.266 (0.910,1.762)	0.161	1.248 (0.895,1.739)	0.191
**Grade**								
G3 vs G1&G2	1.362 (0.941,1.972)	0.102	1.190 (0.806,1.755)	0.382				
T								
T3&4 vs T1&2	1.515 (0.971,2.365)	0.067	1.416 (0.885,2.267)	0.147	3.521 (1.799,6.889)	0.000	3.129 (1.594,6.141)	0.001
N								
N1&2&3 vs N0	1.712 (1.112,2.637)	0.015	1.445 (0.927,2.253)	0.104	2.284 (1.459,3.573)	0.000	2.006 (1.275,3.155)	0.003
M								
M1 vs M0	1.557 (0.810,2.991)	0.184	1.959 (0.996,3.855)	0.051				
**Risk score**	1.689 (1.324,2.155)	0.000	1.785 (1.369,2.329)	0.000	1.313 (1.029,1.675)	0.028	1.359 (1.053,1.753)	0.018

#### Clinical Correlation Analysis of the Prognostic Signature for Gastric Cancer

Risk model illustrated that high-risk score was interrelated to poor prognosis (Figures 5C,E) and histogram showed that Sub B got a higher risk score (Figures 5D,F) in TCGA and GEO cohort simultaneously. The risk curve showed that high risk score was positively correlated with high risk of death (Figure 5G,H). At the same time, the heat map showed that Sub B had a higher risk score, and the expression of the three genes GPC3, GPX3 and PRICKLE1 were higher compared with Sub A in TCGA and GEO cohorts (Figures 5G,I). The ROC curve showed that the risk prediction model had higher specificity and sensitivity when combining with TNM stage in predicting the death of patients (Figure 5K and M). The higher the patient’s T stage, the higher the corresponding risk score (Figure 5L) in TCGA cohort and no significant differences were found in the GEO data (Figure 5N).

#### Analysis of Key Genes Expression Difference in Pan-Cancer

GPC3, GPX3 and PRICKLE1 were the candidate sites selected in the risk prediction model for OS, which indicated that the genes might have potential roles on the malignant behaviors of gastric cancer. Therefore, we had performed pan-cancer analysis of these three genes to provide evidence for future basic research in this field. As shown in Figure 6A, ONCOMINE database was used to perform pan-cancer analysis on GPC3, GPX3 and PRICKLE1 expression (Figure 6A). The expression of these three genes in tumor tissues was higher than that in adjacent tissues in 20 tumors.

## Discussion

Here, we had defined two subtypes with different clinical and biological characteristics through cluster analysis of gastric cancer transcriptome analysis. The repeatability of the classification was confirmed in an independent GEO validation cohort, and consistent phenotypes were observed. Gastric cancer patients with a better prognosis was characterized by higher expression of proliferation cycle suppression related genes. This classification helps us better classify gastric cancer patients and provided targeted treatment based on specific transcriptome data. Over the past decades, scientists had conducted in-depth research work to identify the underlying molecular mechanisms of gastric cancer, identify its prognostic indicators, and explore potential treatment strategies. Our results not only provide insight into the relationship between the proliferation cycle genome subtypes and postoperative survival rate, but also open up new opportunities for improving the management of gastric cancer.

According to the transcriptome data in TCGA cohort, gastric cancer was clustered into Sub A and Sub B by unsupervised cluster analysis. Principal component analysis can also distinguish Sub A and Sub B well. Kaplan-Meier survival analysis showed that Sub A survives longer than Sub B. There were more Sub B cases in TNM 3/4 compared with Sub A cases. Similar conclusions were also verified in GEO cohort. This showed that the clustered Sub A and Sub B had personalized transcriptome expression components and showed different survival times.

Further GO and KEGG analyses showed that the up-regulated genes of Sub A vs Sub B were mostly concentrated in cell cycle inhibition pathways. GSEA analysis also proved the above conclusions on the trend, although a larger sample sizes was required for verification. These indicated that genes related to the regulation of cell proliferation level cycle played an important part in the occurrence and development of gastric cancer. In addition, GO and KEGG analyses revealed that Sub A had high proliferation-related proteins involved in cell cycle checkpoint, cell cycle G2/M phase transition, negative regulation of nuclear division and regulation of spindle checkpoint. These proteins benefit the survival of gastric cancer patients. We found that Sub B cases with poor results showed an opposite proliferative phenotype in functional annotation. We found that tumor invasiveness and patient survival are more influenced by proliferative cycle nature than by other malignant features of gastric cancer. In the cell cycle-related expression analysis, Sub A expressed more cell cycle inhibitory proteins. The lower proliferation ability of Sub A gastric cancer patients may be important for their survival ability better than Sub B.

In addition, we have identified key different molecules between Sub A and Sub B subtypes that have a greater impact on the prognosis through lasso regression method and univariate and multivariate cox regression analysis. In the end, we screened out 3 molecules, GPC3, GPX3 and PRICKLE1, and constructed a risk scoring model for gastric cancer samples. Survival analysis showed that high expression of these three molecules showed worse prognosis in TCGA and GEO cohorts simultaneously.

Glypican 3, also known as GPC3, is a cancer fetal glycoprotein that is attached to cell membranes via glycophosphatidylinositol anchors. GPC3 can regulate cell proliferation in embryonic mesoderm tissue, as GPC3 gene deletion leads to giant/over growth syndrome, simpson-Golabi-Behmel syndrome (SGBS) (Simpson et al., 1975; Behmel et al., 1984; Ferlini et al., 1984; Pilia et al., 1996; Vuillaume et al., 2019). GPC3 is widely expressed in the placenta, liver, lung, and kidney of embryos. On the contrary, it is difficult to detect in most adult organs (Pellegrini et al., 1998). DNA methylation in the GPC3 promoter region may explain this biological downregulation in adult tissues (Hsu et al., 1997; Huber et al., 1999; Boily et al., 2004). A number of innovative treatments for GPC3 have emerged in recent years. The prognostic significance of serum GPC3 levels and tumor cell GPC3 immunoreactivity in patients with hepatocellular carcinoma has been elucidated. Thus, GPC3 has also attracted attention as a useful biomarker and a new therapeutic target molecule. The main mechanism of anti-GPC3 antibody (GPC3Ab) anticancer cells is antibody-dependent cytotoxicity and/or complement dependent cytotoxicity. Because GPC3Ab is associated with immune responses, regiments of combined immune checkpoint inhibitors have also been investigated. In terms of mechanism, GPC3 may be involved in the regulation of Wnt, hedgehog, bone morphogenetic protein, FGF and other signaling pathways, by which it controls the growth and apoptosis of certain types of cells during development (Paine-Saunders et al., 2000; Midorikawa et al., 2003; Capurro et al., 2008; Iglesias et al., 2008).

Glutathione peroxidase 3, also known as GPx3, is a major extracellular GPx isomer and a major scavenger of reactive oxygen species (ROS) in plasma. Some researches show high GPx3 expression predicted poor prognostic survival. Reduced GPx3 expression inhibited the survival of clonal and unanchored cells and prostate cancer (Yu et al., 2007). In addition, GPx3 is necessary to protect cells from exogenous oxidative damage, as demonstrated by high-dose ascorbic acid therapy. GPx3 is essential for the survival of ovarian cancer cells in the ascites tumor environment and protects against extracellular oxidative stressors, suggesting that GPx3 is an important adaptation for metastasis (Lou et al., 2020). In contrast, GPX3 inhibits tumor progression in some tumors. High GPx3 expression is a potential marker for the diagnosis and prognosis and can inhibit the progression in breast cancer, clear cell ovarian cancer hepatocellular carcinoma, cervical cancer and melanoma patients (Itamochi et al., 2002; Saga et al., 2008; Qi et al., 2014; Zhang et al., 2014; Chen et al., 2016; Lou et al., 2020). The study by Chang et al. provided the first *in vivo* molecular genetic evidence that GPx3 does indeed play a tumor suppressor role in the development of prostate cancer (Chang et al., 2016). In esophageal squamous cell carcinoma, GPx3 inhibits tumor migration and invasion through the FAK/AKT pathway (Zhu et al., 2018). High-grade bladder cancer is associated with low urinary GPx3 levels. GPx3 inhibits tumor invasion by inhibiting the JNK-Cjun-MMP2 pathway in liver cancer (Qi et al., 2016). Many studies have shown that methylation-mediated GPX3 inhibition may have important implications for the pathogenesis of cancer. The results of Chen et al. suggest that GPx3 methylation is associated with chemotherapy resistance in head and neck cancer and can be used as a potential prognostic indicator for head and neck cancer patients receiving cisplatin-based chemotherapy (Chen et al., 2011). GPx3 is also down-regulated in hepatocellular carcinoma and esophageal squamous cell carcinoma through promoter hypermethylation, which may lead to cancer development and progression (He et al., 2011; Cao et al., 2015). Silencing of GPx3 through DNA hypermethylation is associated with lymph node metastasis in gastric cancer and cervical cancer (Peng et al., 2012; Zhang et al., 2014). However, the functional research of GPx3 in gastric cancer needs to be further explored.

Prickle planar cell polarity protein 1, is also known as PRICKLE1. The results of Daulat et al. suggested that up-regulation of PRICKLE1 in basal breast cancer, a subtype characterized by high metastatic potential, is associated with poor metastases-free survival. PRICKLE1 promotes cancer cell transmission through interaction with mTORC2 (Daulat et al., 2016). According to a study, PRICKLE1 expression can be used as an independent prognostic factor, can be in the column chart combined with age and TNM staging, to predict the rate of gastric cancer patients with OS. PRICKLE1 expression is an independent prognostic factor in patients with gastric cancer (Zhao et al., 2016; Ding et al., 2020).

Because the conclusions and sample cluster analysis were based on only public sequencing data, and our own clinical sample sequencing data will be needed to verify the conclusion and classification standard in the future. At the same time, the conclusion of this topic needs to be verified by multiple centers. Our subjects were mostly white, so it is not known whether these results are suitable for other groups, such as the yellow race. Future studies with more varied samples are needed. The key prognostic molecules screened out were only used for survival analysis and clinicopathological link analysis in TCGA cohort. They are need to be tested and verified in our own samples and gene function also needs to be further studied through basic experiments in the future.

In this study, cluster analysis based on gastric cancer proliferation-related genes were performed for the first time. The major strength of this study was that the clustering results were verified and analyzed in two independent cohorts. Here, a high-risk Sub B gastric cancer subtype that displayed a lower expression of proliferation-related genes was identified. The biological processes of gastric cancer must be understood to facilitate the improvement of clinical treatments.

In summary, our data indicate that transcriptome analysis divides gastric cancer into two different subgroups with different clinical and biological phenotypes. We believe that this classification is meaningful for different treatment strategies and will lead to targeted treatment of patients with gastric cancer.

## Conclusion

In conclusion, two subtypes with different clinical and biological characteristics was identified through cluster analysis of gastric cancer transcriptome data. The repeatability of the classification was confirmed in an independent GEO validation set, and consistent phenotypes were observed. Gastric cancer patients with a better prognosis was characterized by higher expression of proliferation cycle suppression related genes. This classification helps us to better classify gastric cancer patients and provide targeted treatment based on specific transcriptome data.

## Data Availability

The raw data supporting the conclusion of this article will be made available by the authors, without undue reservation.

## Abbreviations

TCGA, The Cancer Genome Atlas; GEO, Gene Expression Omnibus; NCBI, National Center for Biotechnology Information; UCSC, University of California Santa Cruz; DEG, Differentially expressed gene; STAD, Stomach adenocarcinoma; TPM, transcripts per million reads; MAD, Median absolute deviation; PCA, Principal component analysis; FDR, False discovery rate; GO, Gene ontology; KEGG, Kyoto encyclopedia of genes and genomes; GSEA, Gene set enrichment analysis; ROC, Receiver operating characteristic; AUC, Area under curve; OS, overall survival; HR, Hazard ratio; TMB, Tumor burden mutation; H-TMB, High tumor burden mutation; L-TMB, Low tumor burden mutation.
