# Supplementary figures and images for: Proliferation Cycle Transcriptomic Signatures are Strongly associated With Gastric Cancer Patient Survival
Source: Front Cell Dev Biol. 2021 Dec 1;9:770994. doi: 10.3389/fcell.2021.770994 (PMC8672820; doi:10.3389/fcell.2021.770994)

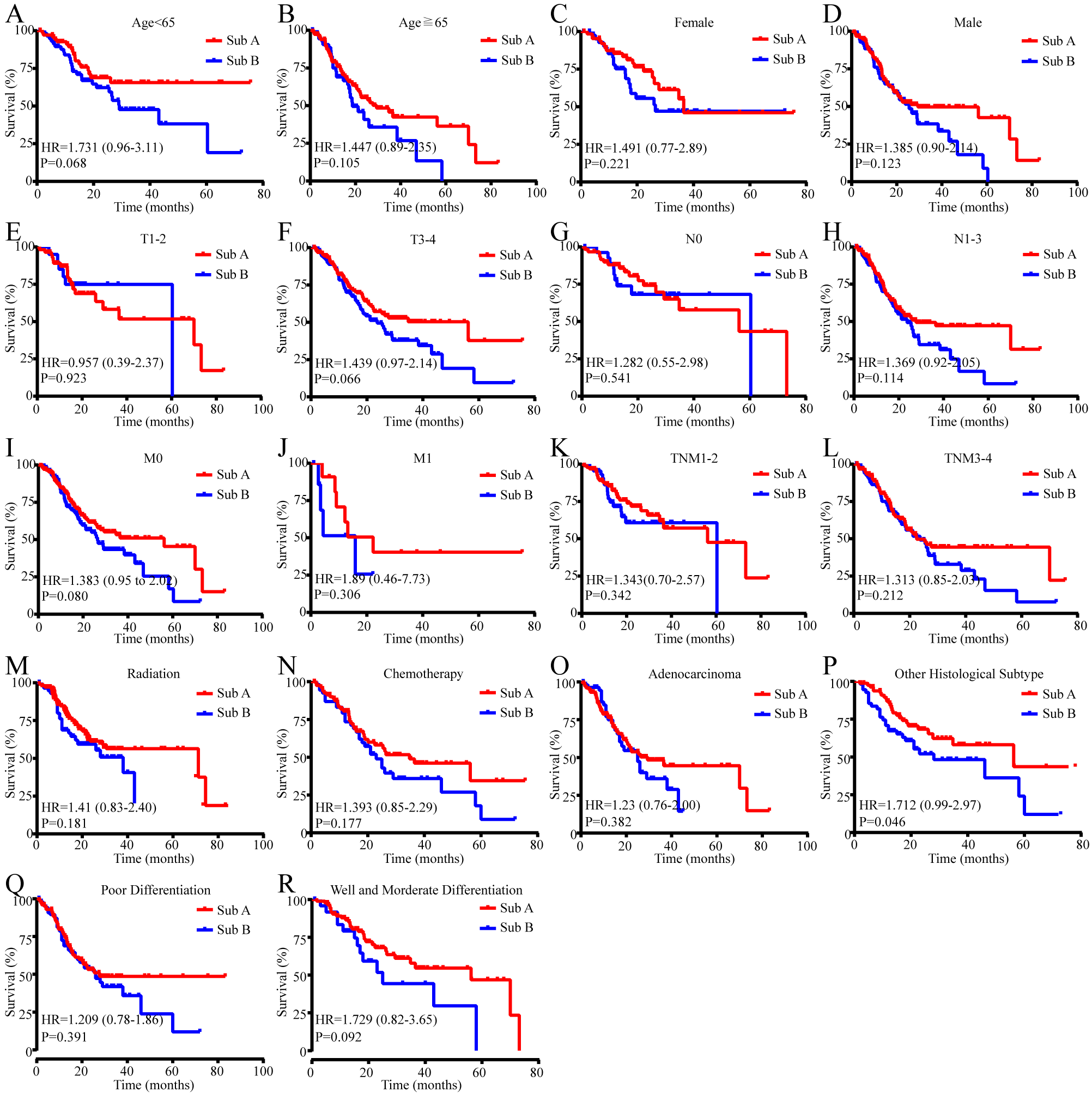

Supplement: Supplementary file 1 [file Image6.TIF]

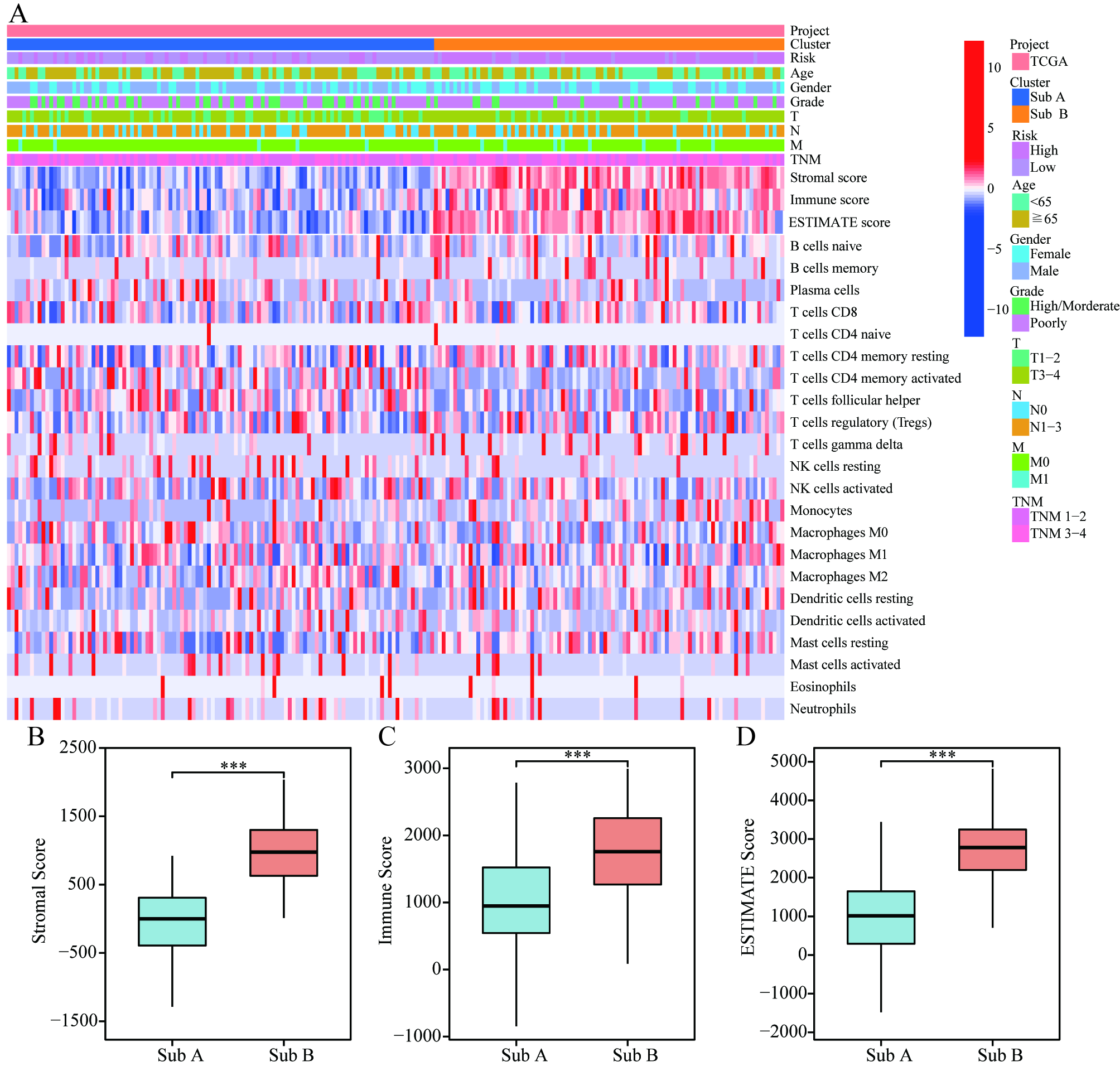

Supplement: Supplementary file 2 [file Image3.TIF]

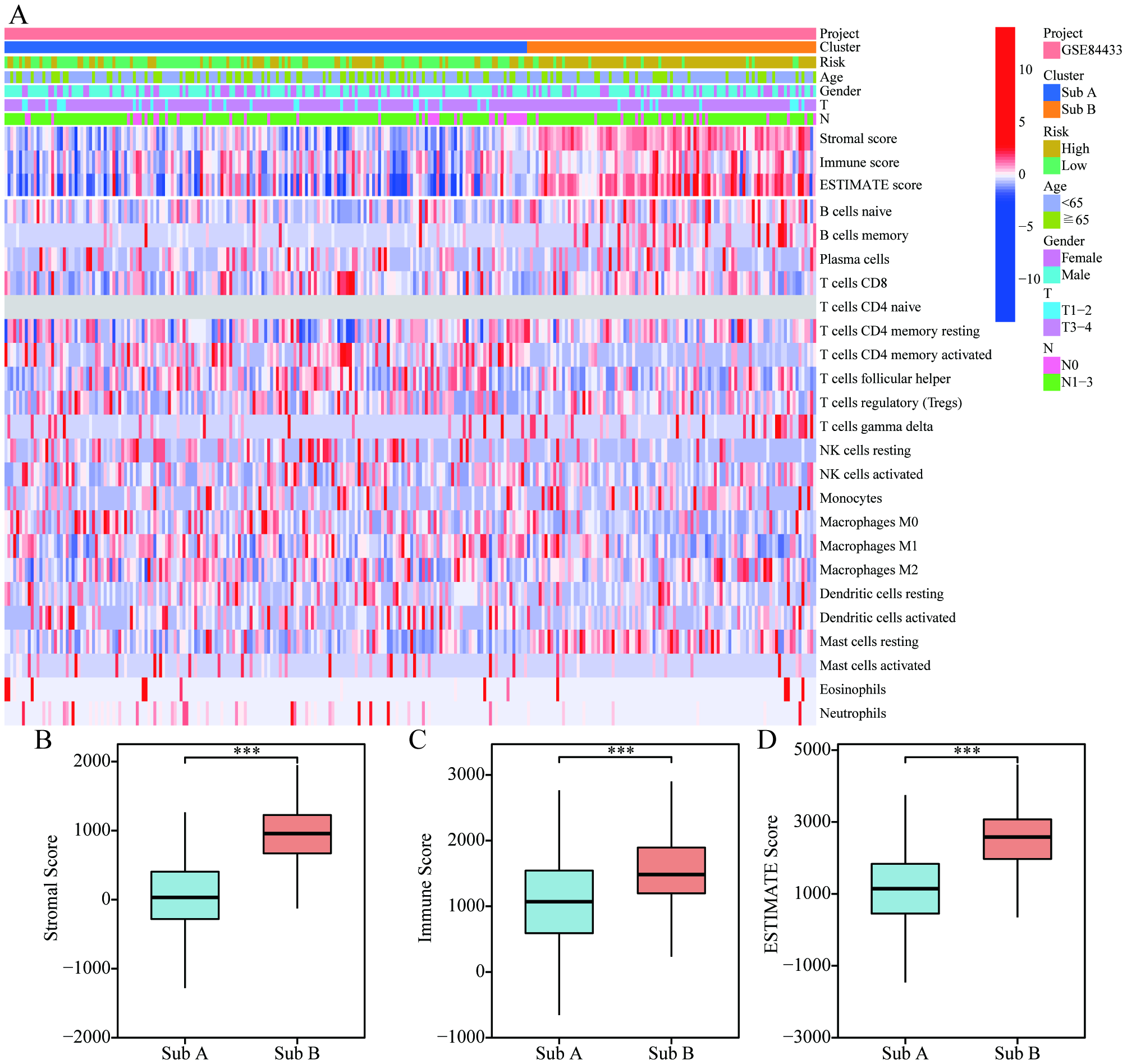

Supplement: Supplementary file 3 [file Image4.TIF]

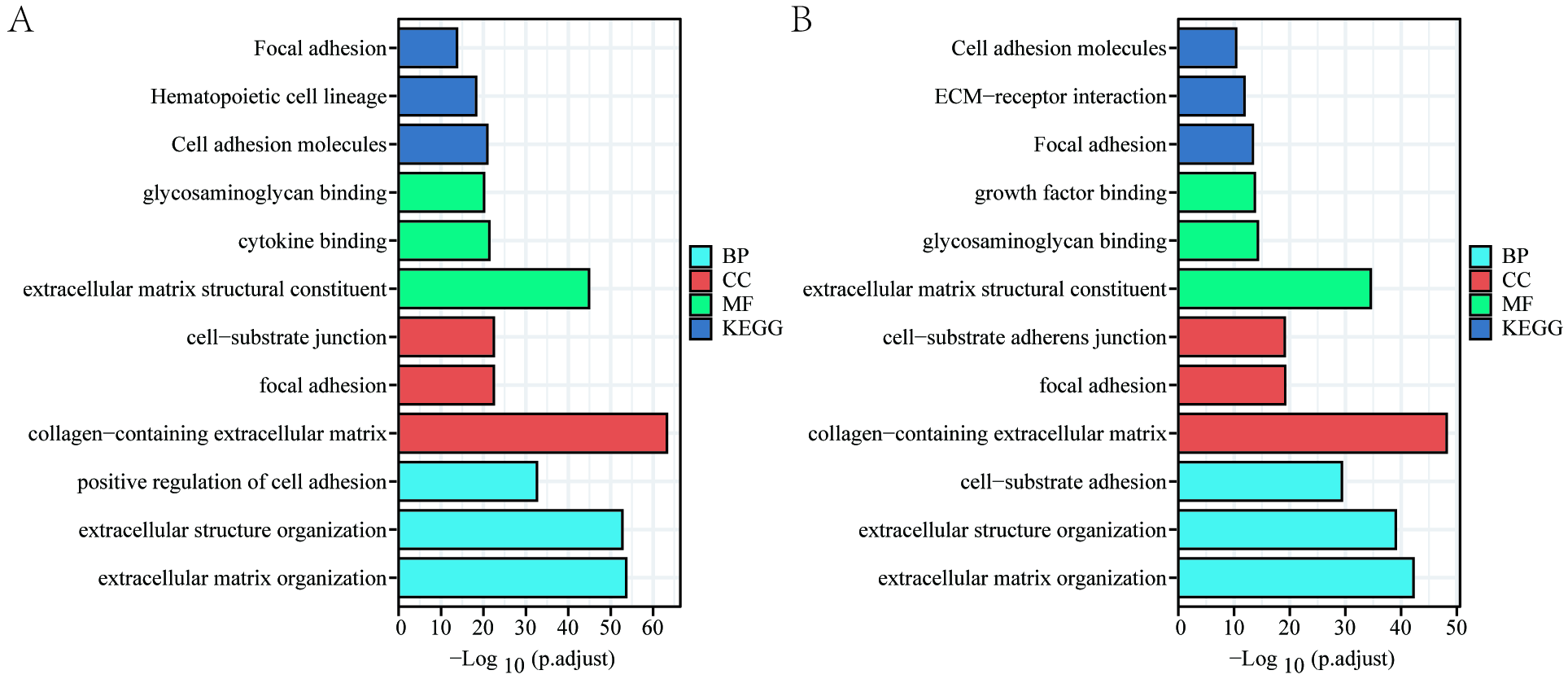

Supplement: Supplementary file 4 [file Image2.TIF]

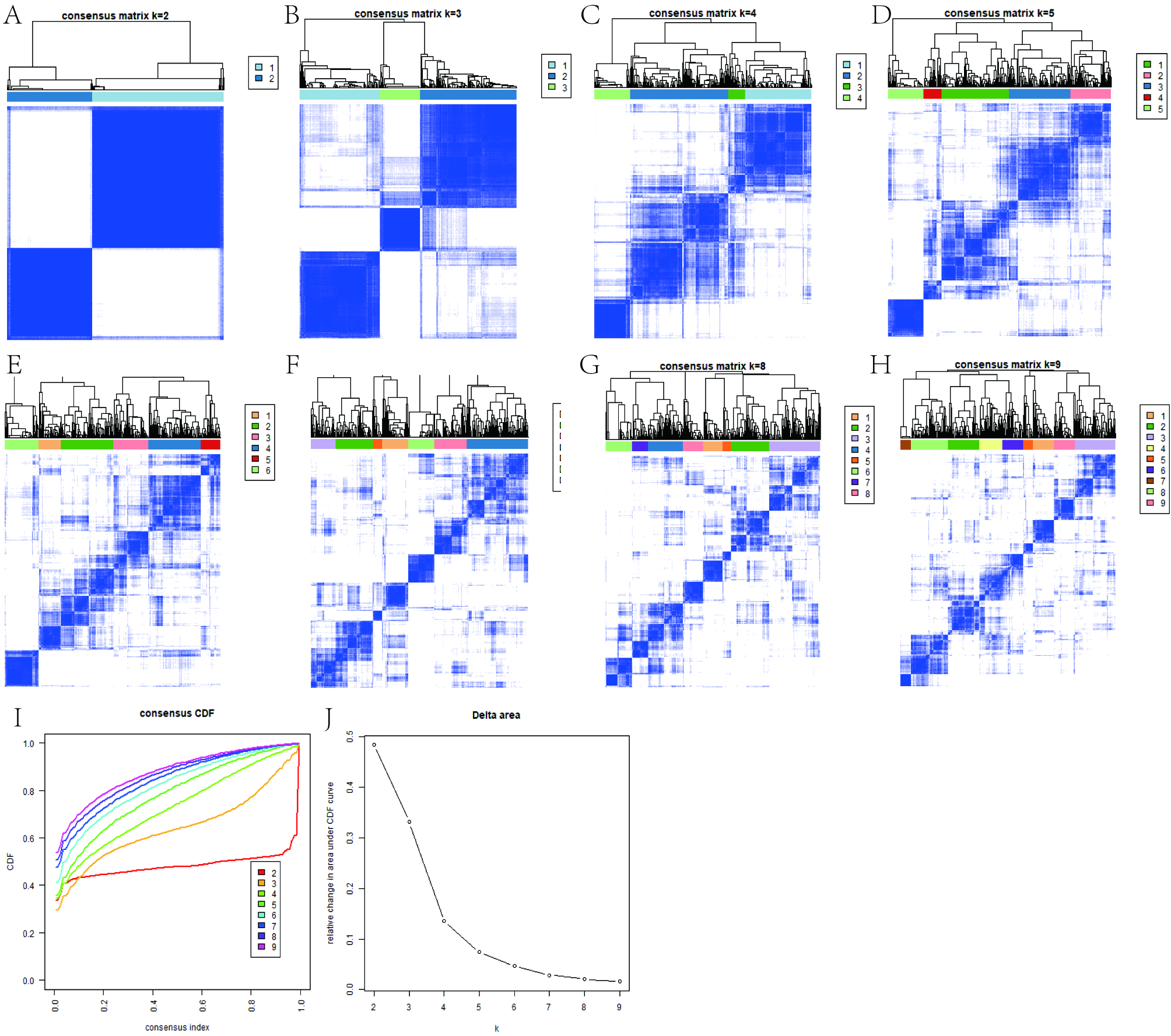

Supplement: Supplementary file 5 [file Image1.TIF]

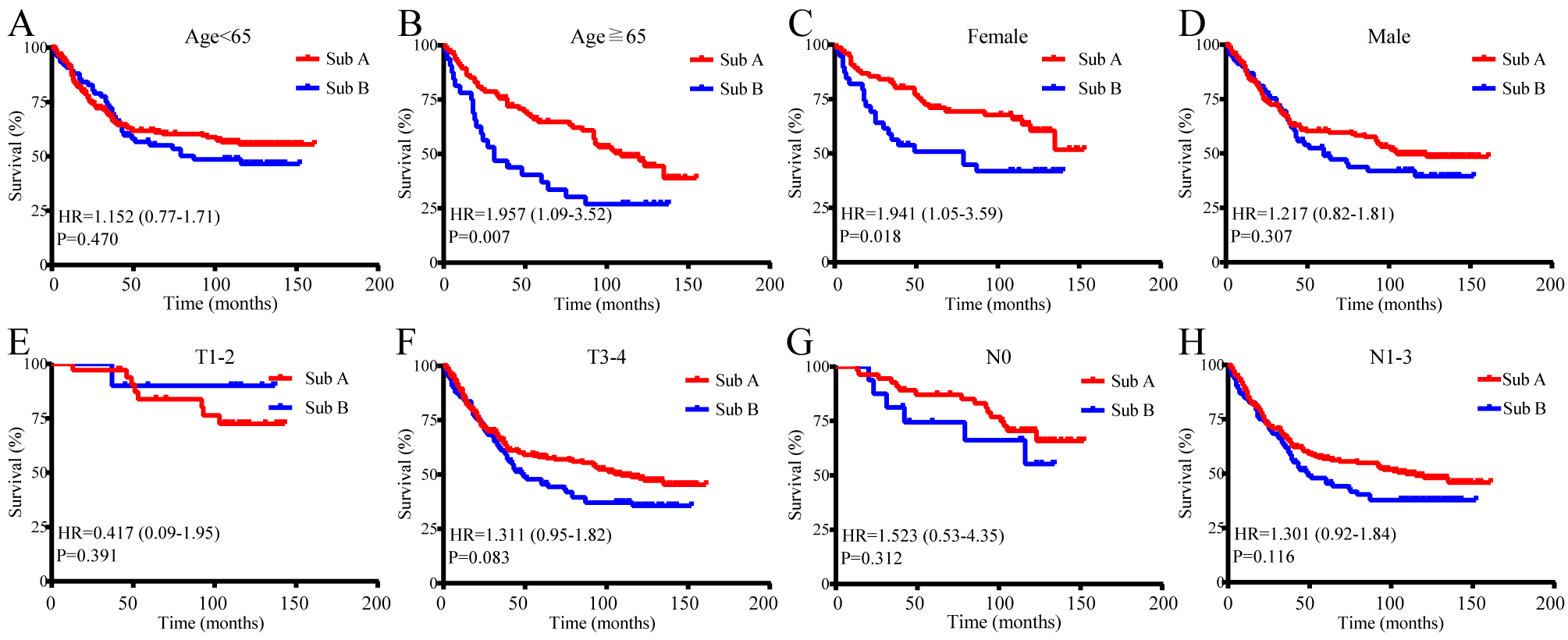

Supplement: Supplementary file 6 [file Image7.TIF]

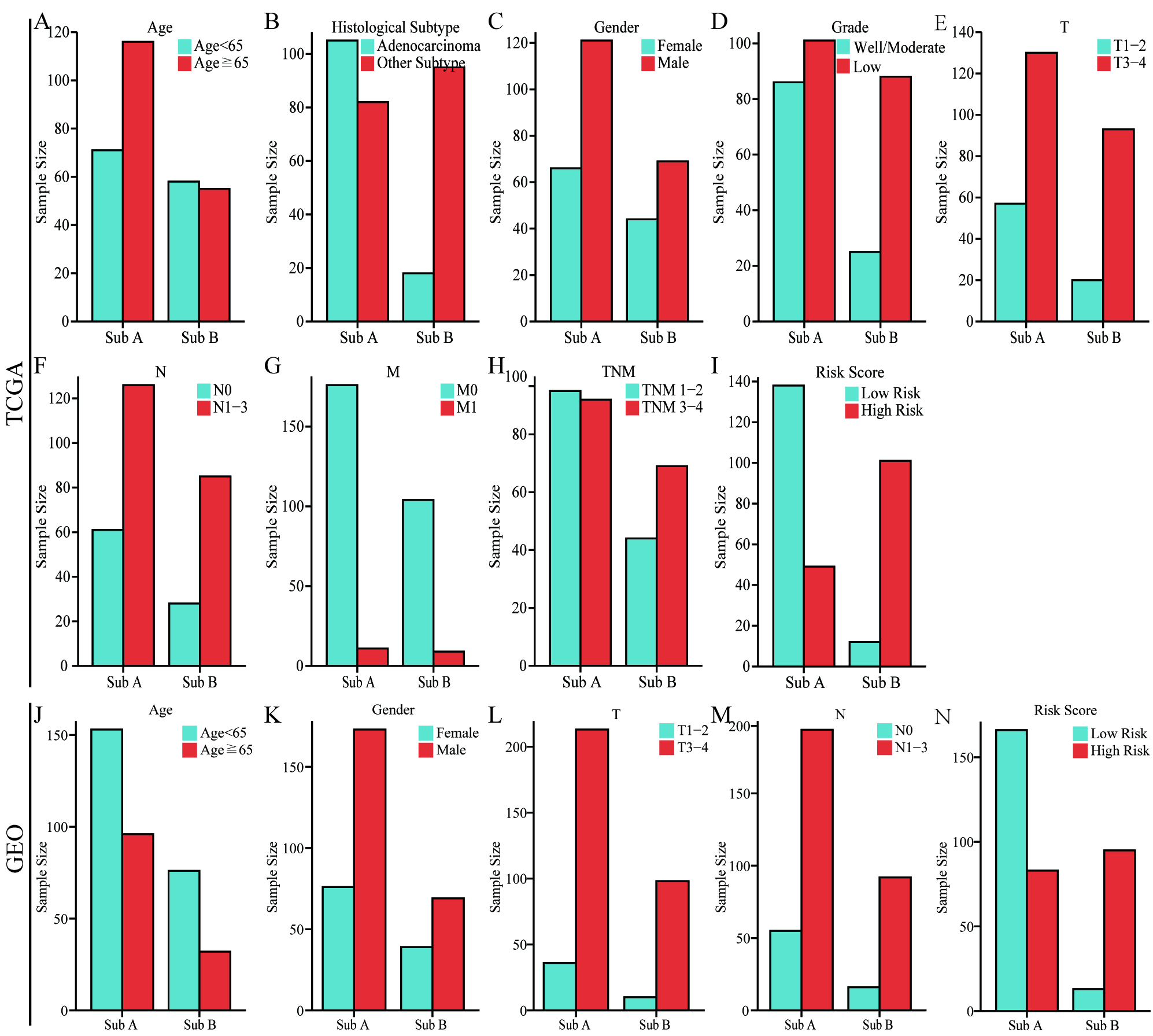

Supplement: Supplementary file 7 [file Image8.TIF]

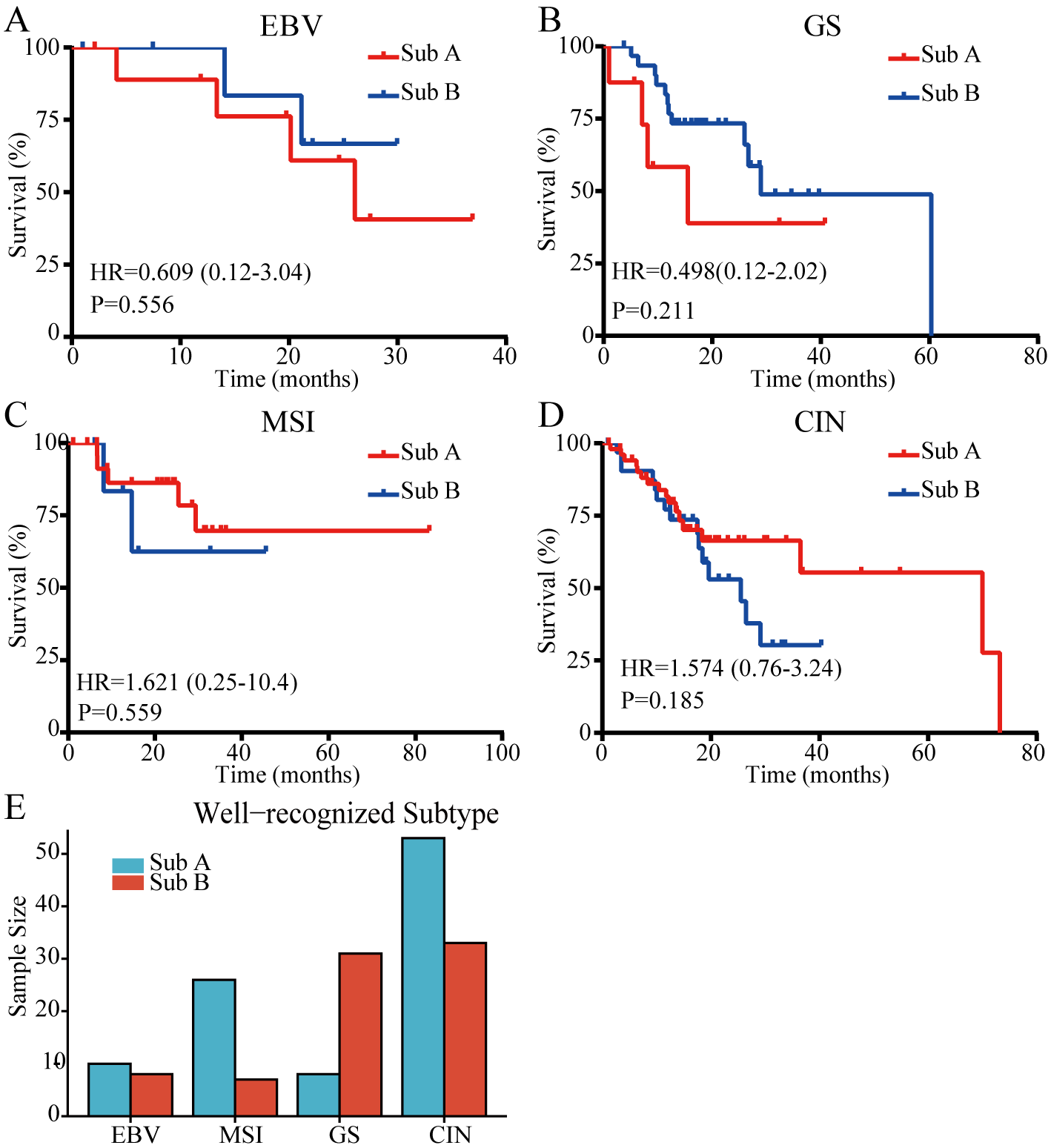

Supplement: Supplementary file 8 [file Image5.TIF]
